# Are overweight and obesity risk factors for invasive mechanical ventilation in severe coronavirus disease 2019 pneumonia?

**DOI:** 10.20945/2359-3997000000350

**Published:** 2021-04-12

**Authors:** Maria Fernanda Coss-Rovirosa, Mercedes Aguilar-Soto, Dalia Cuenca, Mariana Velez-Pintado, Antonio Camiro-Zuñiga, Aldo Ferreira-Hermosillo, Moises Mercado

**Affiliations:** 1 American British Cowdray Medical Center Department of Internal Medicine Mexico City Mexico Department of Internal Medicine, American British Cowdray Medical Center, Mexico City, Mexico; 2 Instituto Mexicano del Seguro Social Centro Médico Nacional Siglo XXI Hospital de Especialidades Mexico City Mexico Research Unit in Endocrine Diseases, Hospital de Especialidades, Centro Médico Nacional Siglo XXI, Instituto Mexicano del Seguro Social, Mexico City, Mexico

**Keywords:** Overweight, obesity, COVID-19, invasive mechanical ventilation (IMV)

## Abstract

**Objective:**

Describe the demographic, clinical, and biochemical characteristics of overweight or obese people with severe COVID-19 pneumonia and evaluate its association with mechanical ventilation requirements in a Mexican cohort.

**Subjects and methods:**

Data were obtained from medical electronic records. Patients were divided in three groups according to the World Health Organization (WHO) classification of body mass index (BMI): lean, overweight and obese. Baseline characteristics and clinical course were compared among these 3 groups.

**Results:**

The study included a total of 355 patients with confirmed COVID-19 diagnoses. Patients with obesity and overweigh, according to the WHO classification, had no significantly increased risk of requiring intubation and invasive mechanical ventilation (IMV) compared to lean subjects, with an odds ratio (OR) of 1.82 (95% CI, 0.94-3.53). A post hoc and multivariate analysis using a BMI > 35 kg/m^2^ to define obesity revealed that subjects above this cut off had as significantly increased risk of requiring IMV after with an OR of 2.86 (95% CI, 1.09-7.05).

**Conclusion:**

We found no higher risk of requiring IMV in patients with overweight or obesity while using conventional BMI cutoffs. According to our sensitivity analyses, the risk of IMV increases in patients with a BMI over 35 kg/m^2^.

## INTRODUCTION

In December 2019, the new coronavirus SARS-CoV-2 was described for the first time in China's Wuhan province ( 1 ). In under 4 months, the virus caused the greatest pandemic of the century.

By September 27, 2020, the World Health Organization (WHO) had reported nearly 33 000 000 cases and over 900 000 deaths worldwide most of them on the American continent. Mexico has reported 817 000 cases since its first case in February 2020 and a mortality rate of 11%, higher than the worldwide average ( 2 ).

An age over 65 years and the presence of comorbidities are the main risk factors for hospitalization ( 3 ). Diabetes, hypertension, and obesity comprise the most common preexisting conditions ( 4 ). Obesity, defined as a body mass index (BMI) of > 30 kg/m^2^, commonly appears in COVID-19 patients, with a 47% prevalence in patients requiring hospitalization ( 5 ). Previous studies demonstrate that patients with obesity have a high risk for a severe disease. These patients often require invasive mechanical ventilation (IMV) and have a higher mortality rate than non-obese patients. In fact, a one-unit increase in BMI likely increases the risk of severe disease by 12% ( 6 ). One cohort study showed that obesity (BMI ≥ 30 kg/m^2^) is a strong, independent, and clinically relevant risk factor for severe illness leading to intubation or death ( 7 ).

In Mexico, obesity's prevalence has risen substantially in the last 30 years and now affects over 35% of the adult population ( 8 ). Obesity associates with other conditions, including cardiovascular disease, type 2 diabetes mellitus, cancer, obstructive sleep apnea, and more ( 9 ). Aside from these metabolic complications, patients with obesity have a chronic inflammatory state ( 10 ). mediated by the adipose tissue's secretion of proinflammatory cytokines, such as adiponectin, interleukin-6 (IL-6), interleukin-1, and tumor necrosis factor-α.

Thus, patients with obesity have an altered innate immune response that negatively affects COVID-19 outcomes ( 11 ). We describe the demographic, clinical, and biochemical characteristics of patients with obesity and overweight and evaluate the association with an IMV requirement.

## SUBJECTS AND METHODS

### Study design and setting

From March 12 to July 15 of 2020, we collected information from patients admitted to the American British Cowdray Medical Center, a private teaching hospital in Mexico City. Clinical, biochemical, and imaging information was obtained and analyzed retrospectively. We included patients 18 years old or older who had a documented diagnosis of COVID-19 (defined as a positive PCR for SARS-CoV2 or a chest CT scan showing characteristics of COVID-19 pneumonia). Data came from the hospital's electronic medical records. We included all patients with height, weight, and outcome data; we excluded patients with missing values. The scientific and ethics committees of the medical center approved the protocol, and the study followed the principles of the Helsinki declaration.

### Study procedures

As stated before, a COVID-19 diagnosis was defined as a positive PCR for SARS-CoV2 or a chest CT scan showing characteristics of COVID-19. We obtained samples for SARS-CoV-2 testing according to the Centers for Disease Control guidelines. Nucleic acid was extracted with the RNeasy Mini Kit (Qiagen), and we used a 7500 Real-Time PCR System (Applied Biosystems) targeting the *CoV E* gene and the *CoV RdRP* gene for nucleic acid amplification. Positive chest CT scans occurred when characteristics of COVID-19 were verified by a consensus among several radiologists.

Patients were classified according to the WHO classification of overweight and obesity. A normal BMI is between 18.5 and 24.9 kg/m^2^, an overweight BMI ranges from 25-29.9 kg/m^2^, and obesity BMI is > 30 kg/m^2^. The main outcome was mechanical ventilation requirement at admission or during hospitalization, as recorded in electronic medical records. We also evaluated days of hospital stay as a secondary outcome.

### Statistical analysis

Depending on data distribution (via the Shapiro Wilks test), continuous variables are described as means with standard deviations (normally distributed) or as a median interquartile range (abnormally distributed). We used frequencies and simple proportions to summarize categorical variables. We used ANOVA to compare groups with normal BMI patients, patients with overweight, and patients with obesity. We conducted univariate, age- and sex-adjusted, and multivariate logistic regressions to evaluate the need for IMV in normal, overweight, and patients with obesity. We also adjusted for possible confounders, such as C-reactive protein levels, oxygen-saturation levels, and mean arterial pressure on admission. We conducted a linear regression analysis using continuous BMI to assess the days of a hospital stay. The same covariates were used for the logistic regression analysis.

Few patients had a normal BMI, so we conducted a sensitivity analysis defining the reference group as patients with a normal or overweight BMI. We also conducted a sensitivity analysis, defining obesity as a BMI over 35 kg/m^2^. All statistical tests were 2-sided, and a p-value < 0.05 was considered statistically significant. We used SAS University Edition version 9.4 statistical software (SAS Institute, Cary, NC).

## RESULTS

This study included a total of 355 patients with confirmed COVID-19 diagnoses. Table 1 depicts the baseline characteristics of patients, who were divided in three groups according to their BMI. Of the patients, 23% (n = 82) had normal BMI, 45% (n = 160) were overweight, and 32% (n = 113) were patients with obesity. The median BMI for each group was 23.4 (22.2-24.4) kg/m^2^, 27.4 (26.1-28.4) kg/m^2^, and 33.7 (31.6-36.4) kg/m^2^, respectively. The mean age was significantly lower (p < 0.0001) in patients with obesity (50.8 ± 13.2 years) compared to those with a normal BMI (56.8 ± 17.3 years). The normal BMI group had a higher proportion of women (40%), with lower percentages of women for overweight (34%) or obesity (29%).

**Table 1:** Baseline characteristics of patients divided by BMI classification

			Categories of weight			p value
			Normal (n=82)	Overweight (n=160)	Obesity (n=113)	
BMI			23.4 (22.2-24.4)	27.4 (26.1-28.4)	33.7 (31.6-36.4)	<0.0001
Age, years			56.8 (17.3)	53.3 (15.7)	50.8 (13.2)	<0.0001
Women n, (%)			33 (40)	54 (34)	33 (29)	0.28
Prediabetes n, (%)			1 (2)	9 (7)	5 (5)	0.27
Diabetes n, (%)			15 (19)	25 (16)	21 (19)	0.83
Hypertension n, (%)			21 (26)	42 (27)	36 (32)	0.53
Severity Scales						
	NEWS (11)		5 (4-7)	6 (4-7)	6 (5-8)	0.11
	MULBSTA (12)		7 (5-10)	9 (5-11)	7 (5-11)	0.67
	CALL-SCORE (13)		7 (5-10)	7 (6-10)	7 (6-10)	0.79
Laboratory values						
	Leucocytes (10^3/uL)		6 (5-9)	7 (5-10)	6 (5-8)	0.03
	Neutrophils (10^3/uL)		5 (3.1-7.5)	5.9 (3.7-8.3)	5 (3-7)	0.18
	Lymphocytes (10^3/uL)		1.0 (0.76-1.37)	0.99 (0.66-1.46)	1.02 (0.80-1.4)	0.75
	Neutrophil/Lymphocyte Ratio		4.8 (2.7-8)	5.6 (3-9.4)	4.8 (2.8-7.7)	0.35
	Glucose (mg/dL)		115 (99-131.8)	116 (98.4-134.9)	114 (97-132)	0.77
	HDL-c (mg/dL)		33.5 (19-40)	35 (28-42)	31 (27-39)	0.256
	DL-c (mg/dL)		58.5 (31-79)	66.5 (47.5-83)	69 (52-93)	0.03
		Triglycerides (mg/dL)	156 (80-156)	126 (95-176)	130 (100-183)	0.018
	CRP (mg/dL)		10.5 (4-17.5)	12 (6-22)	11 (4-20)	0.27
	Ferritin (ng/mL)		762 (337-1385)	897 (435-1623)	1075 (665-1602)	0.11
	IL-6 (pg/mL)		25.5 (16.5-54.5)	38 (16-67)	39 (15-63)	0.57
	D-dimer (ng/mL)		1000 (760-2320)	790 (529-1219)	796 (505-1256)	0.11
Treatment						
	Lopinavir/Ritonavir n, (%)		39 (49)	109 (68)	68 (61)	0.01
	Azithromycin n, (%)		58 (72)	117 (74)	92 (82)	0.17
	Hidroxicloroquine n, (%)		63 (72)	126 (80)	92 (81)	0.31
	Glucocorticoids n, (%)		6 (7)	13 (8)	5 (4)	0.35
	Tocilizumab n, (%)		21 (27)	66 (44)	48 (46)	0.02
Outcomes						
	Required ICU n, (%)		9 (13)	24 (16)	24 (22)	0.15
	Admitted to ICU n, (%)		31 (38)	56 (35)	37 (33)	0.26
	Required mechanical ventilation n, (%)		22 (27)	56 (35)	43 (38)	0.25
	Death (%)		7 (9)	13 (8)	4 (4)	0.25

1 Values are percentages, mean ± SD or median (IQR) as appropriate HDL-c = High density lipoprotein, LDL = Low density lipoprotein, CRP = C-reactive protein, IL-6 = Interleukin 6.

The prevalence of prediabetes (2% of normal BMI patients, 7% of overweight patients, and 5% of patients with obesity, p=0.27), diabetes (normal BMI = 19%, overweight = 16%, obese = 19%, p = 0.83) and hypertension (normal BMI = 26%, overweight = 27%, obese = 32%, p = 0.53) was similar among all groups. Other important comorbidities had a prevalence of 2% for chronic kidney disease, 2.4% for heart disease, and 2% for chronic obstructive pulmonary disease without significant differences among groups (p = 0.38, 0.6, and 0.37, respectively). Cancer's prevalence differed significantly, with 9% in lean patients, 1% in patients with overweight and 4% in patients with obesity (p = 0.015). No between-group difference in severity existed according to validated scores, such as the National Early Warning Score ( 12 ) (p = 0.11), MuLBSTA ( 13 ) (p = 0.67), and CALL ( 14 ) (p = 0.79) scores. Laboratory data were obtained on admission for all values for all patients. The normal BMI and patients with obesity groups had lower leucocyte counts compared to the overweight group (p = 0.03). The groups had similar levels of other inflammatory markers (lymphocyte count, D Dimer, C reactive protein, ferritin, and interleukin-6).

The 3 groups had similar HDL-cholesterol levels, but patients with obesity had higher LDL-c levels. Patients with normal BMI had higher triglyceride levels compared to those who were overweight or obese. All patients were treated with supplemental oxygen by regular nasal cannula or mask upon admission to the hospital. Oxygen was administered via high-flow nasal cannula to 17% of lean individuals, 48% of patients with overweight and 36% of patients with obesity (p = 0.30). Continuous positive airway pressure was used for 5 patients with overweight and 5 patients with obesity. The revised version of the manuscript specifies those details. Secondary infections did not differ between the 3 groups. Bacterial pneumonia was diagnosed in 8% of the patients, without a difference among groups (p = 1). Invasive pulmonary aspergillosis had an overall incidence of 4.2% and was similar between groups (p = 0.33). No significant differences exist in ICU admission, the need for mechanical ventilation, or mortality rate (6% in the general group). The median number of days from symptom onset to hospitalization was 8.5 (6-12, p = 0.33).

Compared to patients with normal BMI, patients with overweight and obesity were more likely to require IMV, but the risk only remained significant for patients with obesity when adjusting for age and sex (OR 1.97, 95% CI, 1.02-3.79). The risk was no longer significant in the multivariate analysis (OR 1.82, 95% CI, 0.94-3.53; Table 2 ).

**Table 2:** Risk for requiring mechanical ventilation among patients with overweight or obesity 1

Normal (n=89)	Overweight (n=160)	Obesity (n=114)
Reference	1.47 (0.82-2.6)	1.7 (0.90-3.1)
Reference	1.63 (0.88-3.01)	1.97 (1.02-3.79)
Reference	0.67(0.29-1.53)	1.82 (0.94-3.53)

1 Values are Odds Ratio (95% CI) unless otherwise specified.

2 Model is multivariate analysis adjusted for age (continuous), sex (men or women), mean arterial pressure on admission (continuous), C reactive protein levels on admission (continuous), oxygen saturation on admission (continuous, percentage).

When conducting sensitivity analyses with normal BMI and patients with overweight together as reference, patients with obesity had a higher risk of requiring IMV and an increased OR after multivariable adjustment (OR 2.04, 95% CI, 1.09-3.81). Considering obesity as having a BMI > 35 kg/m^2^, analyses showed an increased risk of requiring IMV after a multivariate adjustment with an OR of 2.86 (95% CI, 1.09-7.05; Table 3 ).

**Table 3:** Risk for requiring mechanical ventilation considering obesity as BMI >35 kg/m^2^ 1

Without obesity (n=318)	Obesity (n=37)
Reference	1.55 (0.77-3.08)
Reference	2.06 (0.99-4.28)
Reference	2.86 (1.09-7.5)

1 Values are Odds Ratio (95% CI) unless otherwise specified.

2 Model is multivariate analysis adjusted for age (continuous), sex (men or women), mean arterial pressure on admission (continuous), C reactive protein levels on admission (continuous), oxygen saturation on admission (continuous, percentage).

Increased BMI associated with a longer hospital stay according to a bivariate analysis (OR of 1.44), but a multivariate analysis adjusting for age and sex made this association lose statistical significance (OR 1.19). The number of days of a hospital stay positively correlated with the days before a negative PCR after the first positive PCR (r = 0.60, p value < 0.0001).

## DISCUSSION

Obesity and COVID-19 coexist as pandemics in 2020, and obesity associates with increases in the severity, the need for mechanical ventilation, and mortality among these patients ( 15 ). In this study, we analyzed a cohort of 355 COVID-19 patients in Mexico City to compare the clinical characteristics and outcomes of lean, patients with overweight and obesity.

The prevalence of patients with overweight and obesity in our study was 31% and 45%, respectively, and only 24% of patients had a normal BMI. A different cohort of COVID-19 patients in Mexico reported the prevalence of patients with overweight and obesity as 34.9% and 35.9%, respectively ( 16 ). The prevalence of patients with obesity and overweight in Mexico's general population, according to recent data, is 36.1% and 39.1%, respectively, which aligns with data in our cohort ( 7 ).

We divided our cohort into 3 groups according to WHO definitions: normal, overweight, and obesity (BMI > 30 kg/m^2^). We found no significant association between patients with overweight or with obesity and a higher risk of requiring IMV. However, when conducting sensitivity analyses defining obesity as having a BMI > 35 kg/m^2^, we found a higher risk of IMV (OR 2.86, 95% CI, 1.09-7.5). Overall, patients in the 3 groups received most treatments with similar frequencies. However, tocilizumab and lopinavir/ritonavir were more frequently prescribed for patients with overweight and obesity. The decision to initiate each treatment was based on the criteria of the attending physician, but none of these treatments impacted final outcomes ( 17 , 18 ).

Similar findings showed obesity as a risk factor for developing severe COVID-19, with the greatest impact in patients with a BMI ≥ 35 kg/m^2^. In addition, 90% of this group of patients needed IMV ( 5 ). In a similar study in California, a BMI cutoff of > 40 kg/m^2^ showed a greater risk of death after adjusting for obesity-related comorbidities (RR = 2.68, 95% CI, 1.43-5.04) ( 19 ).

These findings support that class II obesity (according to the WHO classification) constitutes a risk factor for needing IMV, but not for patients with overweight or those with class I obesity ( 20 ). Obesity-related comorbidities that might act as confounders (such as diabetes and hypertension) were similar in the three groups and did not associate with IMV in univariate analyses.

Patients with obesity have lower mortality in some chronic diseases. Similarly, patients with obesity have a lower acute respiratory distress syndrome associated mortality than does the general population ( 21 ). This phenomenon – the obesity paradox for chronic diseases – might also affect patients with COVID-19. Nevertheless, this paradox may merely reflect a selection bias ( 22 ). Early reports on COVID-19 suggested patients with overweigh and with obesity had an increased risk of complications, so it may be prudent to hospitalize these patients even if they present with mild disease ( 23 ). Another explanation for why overweight subjects and patients with lower grades of obesity have no higher risk for IMV is the concept of metabolically healthy obese patients. These patients have a preserved insulin sensitivity, a lower liver fat content, lower visceral fat mass, and normal adipose tissue function, conferring them a lower risk of mortality in some chronic diseases. This is further supported because obesity-related comorbidities, such as diabetes and hypertension, were similar in the three groups, as were glucose levels on admission but not for triglycerides and LDL-c levels ( 24 ).

For patients with class II or higher obesity, several mechanisms might increase their risk of mechanical ventilation. Adipose tissue is a proinflammatory tissue with an increased expression of cytokines, particularly adipokines like leptin, which associate with an increased inflammatory response, reduced ciliary clearance, and acute respiratory distress syndrome. A delayed immune response that impairs the response to infectious agents also appears in patients with obesity ( 21 ).

On the other hand, patients with obesity have excess body weight and poor pulmonary reserve, which alters pulmonary gas exchange and respiratory mechanics. This could necessitate early intubation and IMV due to an extremely rapid O2 desaturation, particularly in patients with higher degrees of obesity ( 25 ).

Our results show obesity could constitute an important risk factor for severe diseases requiring IMV, but only in patients with a BMI > 35 kg/m^2^. Other studies found similar results but did not compare categories of patients of overweight or obesity using the conventional BMI cutoff > 30 kg/m^2^. These findings suggest that only higher degrees of obesity increase the risk for IMV and worse outcomes with COVID-19. Not all patients with obesity and overweight might have the same risk, so they should be carefully evaluated to avoid preventive hospitalization when presenting a mild disease.

This study has some limitations, including its retrospective nature and its lack of non-hospitalized patients. However, it represents a tertiary care center's experience, which can support prospective studies around the country.

We conclude that patients with obesity, defined by the WHO as having a BMI > 30 kg/m^2^, hospitalized for a SARS-CoV-2 infection had a similar risk for IMV compared to lean patients. The increased risk for IMV only appeared in patients with class II obesity.
